# P006. Clinical management of migraine attributed to cerebral venous sinus thrombosis

**DOI:** 10.1186/1129-2377-16-S1-A170

**Published:** 2015-09-28

**Authors:** Rosario Iannacchero, Amerigo Costa, Rita Carlotta Santoro, Antonio Sergi, Aida Squillace, Giuseppe Vescio, Umberto Cannistrà

**Affiliations:** Centre for Headache and Adaptive Disorders, Unit of Neurology, Department of Neuroscience and Sense Organs, Azienda Ospedaliera “Pugliese-Ciaccio”, Catanzaro, Italy; Unit of Hemophilia and Coagulation Disorders, Department of Hematology, Oncology and Transfusion Medicine, Azienda Ospedaliera “Pugliese-Ciaccio”, Catanzaro, Italy; Unit of Diagnostic Radiology, Department of Services, Azienda Ospedaliera “Pugliese-Ciaccio”, Catanzaro, Italy; Department of Health Science, Magna Graecia University, Catanzaro, Italy

## Background

Cerebral venous sinus thrombosis (CVST) is the presence of a blood clot in the dural venous sinuses. Symptoms include migraine, abnormal vision, stroke-like signs, seizures, and abnormalities of consciousness and mental status. CVST incidence is 3-4 cases per million adults with a higher frequency among younger people < 40 years old, thrombophilia patients, women who were pregnant and using hormonal contraceptives. Other risk factors are chronic inflammatory diseases, blood disorders, meningitis, ear, nose and throat infections, venous sinuses injuries, surgical procedures in the head and neck area, homocystinuria. Diagnosis requires neuroimaging. Recommended treatment is with anticoagulants [1]. The aims of our retrospective study were to detect CVST incidence among patients accessing our outpatient headache day service (DS) programme, whose main symptom was headache, to compare data with pre-existing estimation, and to identify diagnostic and therapeutic procedures in our clinical management of CVST.

## Materials and methods

We reviewed records of patients accessing our day service from September 2013 to April 2015 and selected cases that had prompted CVST assessment. We collected demographic information, medical history, clinical, psychosocial and neurocognitive assessments data, neuroimaging, referrals and therapy. We performed descriptive statistics for all data.

## Results

In the population accessing our DS in the considered period (n=320), 13 patients (4.06%; 38.34±7.08 mean age; 4 males; 9 females) underwent a venous magnetic resonance angiography and findings were compatible with CVST. They were referred to the Unit of Hemophilia and Coagulation Disorders to be assessed for thrombophilia. Five headache presentations had a secondary migraine pattern associated with CVST being unresponsive to treatment and steadily worsening. Other clinical signs, symptoms, and medical history cues that prompted CVST assessment were stroke-like indicators (n=3); use of hormonal contraceptives (n=2); homocystinuria (n=1); blood disorder (n=1); chronic inflammatory disease (n=1); surgical procedure in the head area (n=1); cognitive and behavioral abnormal state (n=1). Prothrombin mutation was found in 8 patients. Four patients examined with venous angio-CT received anticoagulant treatment in addition to headache management.

Written informed consent to publish was obtained from the patient(s).

## Conclusions

Our diagnostic and therapeutic headache programme is able to detect and appropriately refer CVST secondary migraine. The incidence of diagnosed CVST is higher than expected by population estimation and we could explain this with the overlap between headache and CVST demographics and risk factors.
